# Maternal antenatal anxiety and electrophysiological functioning amongst a sub-set of preschoolers participating in the GUSTO cohort

**DOI:** 10.1186/s12888-020-2454-3

**Published:** 2020-02-12

**Authors:** Hong Kuang Tan, Shaun K. Y. Goh, Stella Tsotsi, Michaela Bruntraeger, Helen Yu Chen, Birit Broekman, Kok Hian Tan, Yap Seng Chong, Michael J. Meaney, Anqi Qiu, Anne Rifkin-Graboi

**Affiliations:** 1 Integrative Neurosciences, Singapore Institute for Clinical Sciences (SICS), Agency for Science and Technology (A*STAR), Brenner Centre for Molecular Medicine, 30 Medical Drive, Singapore, 117609 Singapore; 2 Duke-National University of Singapore, 8 College Road, Singapore, 169857 Singapore; 3 Department of Biomedical Engineering, National University Singapore, 4 Engineering Drive 3, Singapore, 117583 Singapore; 4 Present Address: Centre for Research in Child Development, National Institute of Education, 1 Nanyang Walk, Singapore, S637616 Singapore; 5 Wellcome Trust Sanger Institute, Wellcome Genome Campus, Hinxton, Cambridge, CB10 1SA UK; 6 Department of Psychological Medicine, KK Women and Children’s Hospital, 100 Bukit Timah Road, Singapore, 229899 Singapore; 7 Department of Psychiatry, OLVG and Amsterdam UMC, Amsterdam, Netherlands; 8 Division of Obstetrics and Gynaecology, KK Women and Children’s Hospital, 100 Bukit Timah Road, Singapore, 229899 Singapore; 9 Department of Gynaecology and Obstetrics, National University Hospital Singapore, 1E, Kent Ridge Road, Singapore, 119228 Singapore; 10 McGill University, 6875 Boulevard Lasalle, Montréal, QC H4H 1R3 Canada; 11 Ludmer Centre for Neuroinformatics and Mental Health, 6875 Boulevard Lasalle, Montréal, QC H4H 1R3 Canada

**Keywords:** Maternal mental health, Executive functioning, Preschool, Memory, Event related potentials (ERP)

## Abstract

**Background:**

Antenatal maternal anxiety is a risk for offspring psychological and cognitive difficulties. The preschool years represent an important time for brain development, and so may be a window for intervention. However, electrophysiological investigations of maternal anxiety and preschoolers’ brain functioning are lacking. We ask whether anxiety symptoms predict neurophysiology, and consider timing specificity (26-weeks antenatal or 24-months postnatal), form of insult (anxiety symptoms, per se, or also depression symptoms), and offspring gender.

**Methods:**

The sample consisted of a subset of 71 mothers and their 3 year old children taking part in the prospective birth cohort, GUSTO. Mothers provided antenatal (26 weeks) and postnatal (2 years) anxiety and depressive symptomatology data, respectively via the “State Trait Anxiety Questionnaire” and the “Edinburgh Postpartum Depression Scale.” Offspring provided electrophysiological data, obtained while they indicated the emotional expression of actors whose facial expressions remained consistent throughout a pre-switch block, but were reversed at “post-switch.”

**Results:**

Three electrophysiological components linked to different information processing stages were identified. The two earliest occurring components (i.e., the N1 and P2) differed across blocks. During post-switch, both were significantly predicted by maternal anxiety, after controlling for pre-switch neurophysiology. Similar results were observed with depression. Antenatal mental health remained a significant predictor after controlling for postnatal mental health.

**Conclusion:**

In combination with past work, these findings suggest the importance of reducing symptoms in women prior to and during pregnancy, and offering support to offspring early in development.

## Background

Roughly 25% of women in their second trimester of pregnancy indicate some anxiety symptoms and roughly 15% of pregnant women meet clinical criteria [1]. Yet, the number of indirectly affected individuals is likely greater-- antenatal maternal anxiety is related to a variety of offspring developmental outcomes. At age 18, offspring of mothers who had been anxious during pregnancy and were taking part in the ALSPAC cohort study had a 1.39 increased Odds Ratio of being diagnosed with anxiety [2]. In earlier life, antenatal anxiety relates to parent and/or teacher reported preschool problematic behavior such as inattention or conduct problems, emotional symptoms, and/or comparatively poor cognitive development/inattention [3–5].

In keeping with the recognition that child cognitive factors may moderate associations between risk and psychopathology (e.g., [6]), research examines the association between maternal anxiety and offspring lab-based cognitive functioning, including executive control. For example, antenatal anxiety associates with offspring school aged and adolescent working memory, cognitive inhibition, and executive functioning [7–10]. Limited lab-based work with younger children reports similar associations: high antenatal maternal anxiety relates to reaction time variability, a potential marker of intelligence or attentional difficulties, amongst 5 year olds [4].

Furthermore, antenatal maternal anxiety is found to predict offspring brain functioning, as measured by Event Related Potentials (ERP’s). ERP amplitudes indicate the degree of coordinated neuronal activity in response to stimuli presentation [11]. ERP’s are not only a non-invasive way of examining brain functioning, but also reveal cognition as it unfolds. Such precision may be useful in tailoring offspring-directed prevention programs to fit individual cognitive-emotional difficulties. As reviewed by Pires et al. [12] the N1, which occurs within the first 200 msec post-stimulus, may reflect inhibition at the sensory or exogenous level; the P2, which occurs following the N1 but still generally within 200 msec post-stimulus [12] is commonly associated with early attention and perception of emotion [13–15]; and the later appearing N2 negative deflection, often occurring between 200 and 400 msec post-stimulus, is often linked to top-down inhibitory/executive control rather than response to stimulus discrepancy [16, 17].

Less work examines these components in preschoolers, though in conjunction with studies of slightly older young children, existing research suggests that these components are evoked in executive functioning tasks and may reflect somewhat similar, but not identical, processes. For example, when children between roughly 5 to 8 years of age took part in a cued-switch task, Elke & Wiebe [18] observed stimulus-locked P2’s, though switch-related amplitude differences were only observed in children in the older age ranges [18]. With regards to the negative deflections, within an examination of both adults’ and children’s ERP responses to conflict, N1 and N2 components were observed in both age groups, with the N1 and N2 more pronounced in the children [19]. However, congruent versus incongruent stimuli only elicited marginal differences in the N1 in children, and neither group exhibited differences in the N2 by stimuli type [19]. Somewhat similarly, results from a different Flanker study performed with children aged 4–8 suggests that the N2 can be observed regardless of age, and that its amplitude to incongruent stimuli relates to orienting abilities; however, that study also found that differences in N2 amplitudes across congruent and incongruent stimuli were only apparent, and only associated with executive control capabilities, amongst children 6 years and older [20]. Still, other work examining performance during a switch-task in children roughly 3.0–4.5 years of age suggests a role for the N2 in executive control, as amplitudes are smaller amongst those who pass the switch than those who fail [21]. Thus, there is still much to learn with regards to the nature of these components in preschool executive functioning research, and accordingly, their association with potential risk factors such as maternal anxiety.

Amongst adolescents, ERP research has uncovered associations between maternal antenatal anxiety and adolescent offspring inhibitory processing [7] and cognitive evaluation [7, 22], but not stimulus driven inhibition [7]. In contrast, in infancy, associations between maternal antenatal anxiety and earlier occurring, externally driven, aspects of cognitive functioning have been observed [23]. Likewise, studies report associations with early-to-mid occurring components potentially reflecting attention and/or attentional biases [24]. Still, to our knowledge no published work examines antenatal anxiety, or the closely related condition of antenatal depression, and ERPs during preschool, a period of rapid cognitive development when many executive functions beginning to come on-line.

### Maternal “antenatal anxiety”, per se?

Despite research linking antenatal maternal mental health and stressful life experiences to offspring brain development, the precise biological mechanisms remains unclear. Possibilities include growth restriction in otherwise at-risk populations, direct and indirect (e.g., cytokine) influences upon cortisol transfer across the placenta, and moderation by genetic, ethnic, and postnatal environmental factors (e.g., see reviews by [25, 26]). Many of such potential mechanisms are not specific to anxiety, and may also be expected to be influenced by comorbid conditions like depression. Still, some past work may suggest specificity. Anxiety predicted differences in neonatal brain micro-structure are not explained by perinatal depression [27]. Pregnancy specific anxiety, but not state anxiety or depression, negatively impacts inhibitory control [28] at school age.

In addition, given the recognized stability between antenatal and postnatal maternal mental health, it remains possible that “antenatal” effects may, also be explained via postnatal mechanisms [8, 29]. Postnatal maternal mental health may affect offspring development via alterations in parenting practices, which are stress inducing to children and/or limit stimulation and support [30, 31].

### Gender

Offspring gender may moderate associations (e.g., [32]). Females (e.g., [28, 33]) and males (e.g., [9, 34]) have been reported to be more vulnerable to antenatal maternal mental health.

### Study aims

We investigate antenatal maternal anxiety symptoms in relation to 3.5 year-old offspring neurophysiology within a novel ERP task. As this was the first ERP preschool study of its kind, we created a task that, similar to what children experience in the real-world, required the use of a number of processes potentially impacted by anxiety: the perception and exogenous perception of stimuli; attention/perception to emotional faces; endogenous control/inhibition; and memory. Nevertheless, this procedure still allowed us to examine the time course of information processing.

We examined relations between antenatal maternal anxiety symptoms and three stimulus elicited ERP components likely akin to the aforementioned “N1”, “P2” and “N2”, and potentially respectively reflective of exogenous inhibition, early attention/emotional processing, and endogenous inhibition. However because latter components occur, de-facto, after prior components, rather than examining the absolute amplitude of the P2 and N2, instead the “P2_N1” (i.e., P2 minus N1) and “N2_P2” (i.e., N2 minus P2) complexes were examined to isolate unique neural activity at different points in time [35].

We hypothesized a relation between antenatal anxiety and preschooler electrophysiology, but were uncertain whether it would be limited to an association with endogenous inhibition (e.g., the N2_P2), similar to when offspring are at much later stages of development [7], or a more broad array of components, more in keeping with infant research [23]. We additionally explored specificity of timing (antenatal or postnatal) and insult (anxiety symptoms per se or also depression symptoms), as well as the potential moderating role of gender.

## Materials and methods

### Participants

**Fig. 1:**
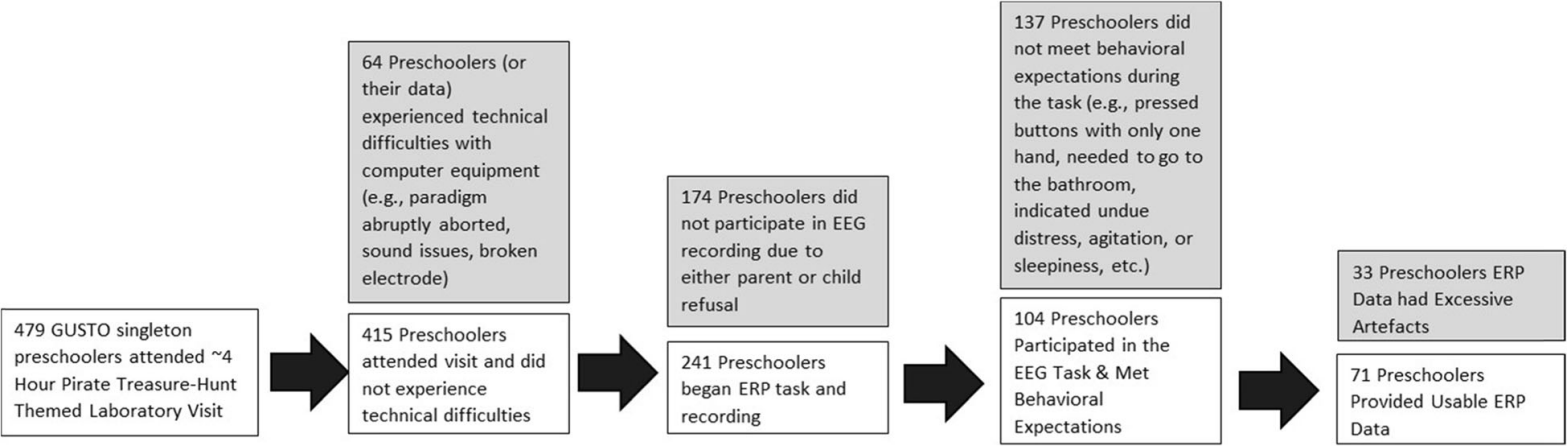
Of the 483 children who participated in a pirate treasure-hunt themed laboratory visit, 479 were singletons and eligible for this study. The entire visit lasted roughly 4 h and included a variety of behavioral, eye tracking, and psychophysiological assessments. The ERP assessment occurred roughly 36 min after arrival, following a task designed to induce joy as well as the placement of heart rate electrodes (data not reported here). Of these participants, 64 experienced technical difficulties with computer or EEG equipment; 174 did not participate in the EEG recording due to either parent or child refusal to take part in the task and/or wear the net either initially or into the post-switch phase; 137 did not meet behavioral expectations (e.g., pressed buttons with only one hand, needed to go to the bathroom, indicated undue distress, agitation, or sleepiness, etc.); 33 had excessive artifacts, and 71 provided usable ERP data (See Results for comparisons of subject characteristics between children providing and not providing artefact-free data)

We focus on data from 71 mother-preschool (male offspring *n* = 42; female offspring *n* = 29) dyads, taking part in the larger prospective birth cohort study – Growing Up in Singapore Toward healthy Outcomes, which initially enrolled pregnant women attending one of two primary birthing hospitals in Singapore (“GUSTO”, [36]). Mothers were assessed from pregnancy and, with their children, a subsample were invited to the Neurodevelopment Research Center when the children were three-and-a-half years of age (see Fig. 1). Eligibility for the current research was limited to those who were singletons at birth (e.g., no twins or triplets). Children were roughly 3.5 years old at the time of testing, with a mean age since delivery of 1257.91 days (SD = 28.36). All participants were Singaporean ethnic Chinese, ethnic Malay, or ethnic Indian. (See “Descriptive statistics and comparisons of included versus excluded dyads” for additional study participant information.)

Maternal demographics (e.g., education, household income, and age) were collected during the antenatal period. Income was assessed in bands, with a score of “4” indicating 4000–5999 SGD and a score of “3” indicating 2000 to 3999 SGD per month. As a point of reference, in 2013 the median Singaporean household income was $6257 SGD [37]. Maternal education was assessed using an ordinal scale. For example, “2” = any secondary school (similar to “any high school”); “3” = GCE A Level or ITE/NTC (somewhat similar to an Associate’s degree); “4” = University degree.

### Maternal mental health

Given our interest in the influence of pre- versus post- natal anxiety exposure, here, we focus on the State scale of the State-Trait Anxiety Inventory (STAI) form Y [38], In the larger GUSTO cohort, the Cronbach’s alphas for the State Scale at these time points were respectively 0.91 and 0.93.

Depressive symptoms were assessed via the Edinburgh Postpartum Depression Scale (EPDS) [39]. In the larger GUSTO cohort, the EPDS Chronbach alphas at these times were, respectively, 0.82 and 0.87.

Mothers received questionnaires at 26 weeks antenatal and 24 month postnatal.

### ERP task design and administration

The ERP recording occurred whilst children were asked to identify, via button press, happy versus angry “pirates.” Pirate stimuli consisted of Nimstim [40] male faces with either happy or angry expressions on a purple or orange background, edited to be wearing a pirate hat. The NimStim database is highly used in developmental research, with Barnard-Brak and colleagues (Barnard-Brak 2017) reporting that over 800 studies of children have used this picture bank. Moreover, in their own research of 167 young children aged 30–83 months, Barnard-Brak et al. [41] found good evidence of reliability and construct validity regardless of race, in a subsample of Nimstim pictures that Tottenham and colleagues [40] reported as receiving a reliability rating of .80 or above. In the current research, chosen NimStim stimuli had received reliability ratings of at least 0.9 for angry expressions and 0.98 for happy expressions [40]. The stimuli were rectangular in shape (length 16.8 cm and height 13.5 cm) and presented in the middle (6.8 cm from the top and bottom and 8.5 cm from the side bezels) of a 17 in., 4:3 dell monitor screen.

In the Pre-Switch condition children were exposed to 50 trials with Pirate A appearing happy on a Color A background and Pirate B appearing angry on a Color B background. In the Post-Switch Blocks the pirates faces were “switched” for 66/78 trials. That is, children viewed the same stimuli, but for the majority of trials Pirate A now appeared angry on his same color background and Pirate B now appeared happy on his same color background, with the exception that in each post-switch block a small proportion of stimuli (12 trials) were identical to those used in the pre-condition trials. These 12 “pre” switch trials were intentionally included in the “post” switch block to maintain their pre-potent influence, prevent a complete association between the new color--expression pairings, and maintain the need for cognitive inhibition/selection during the latter post-switch stages.

**Fig. 2:**
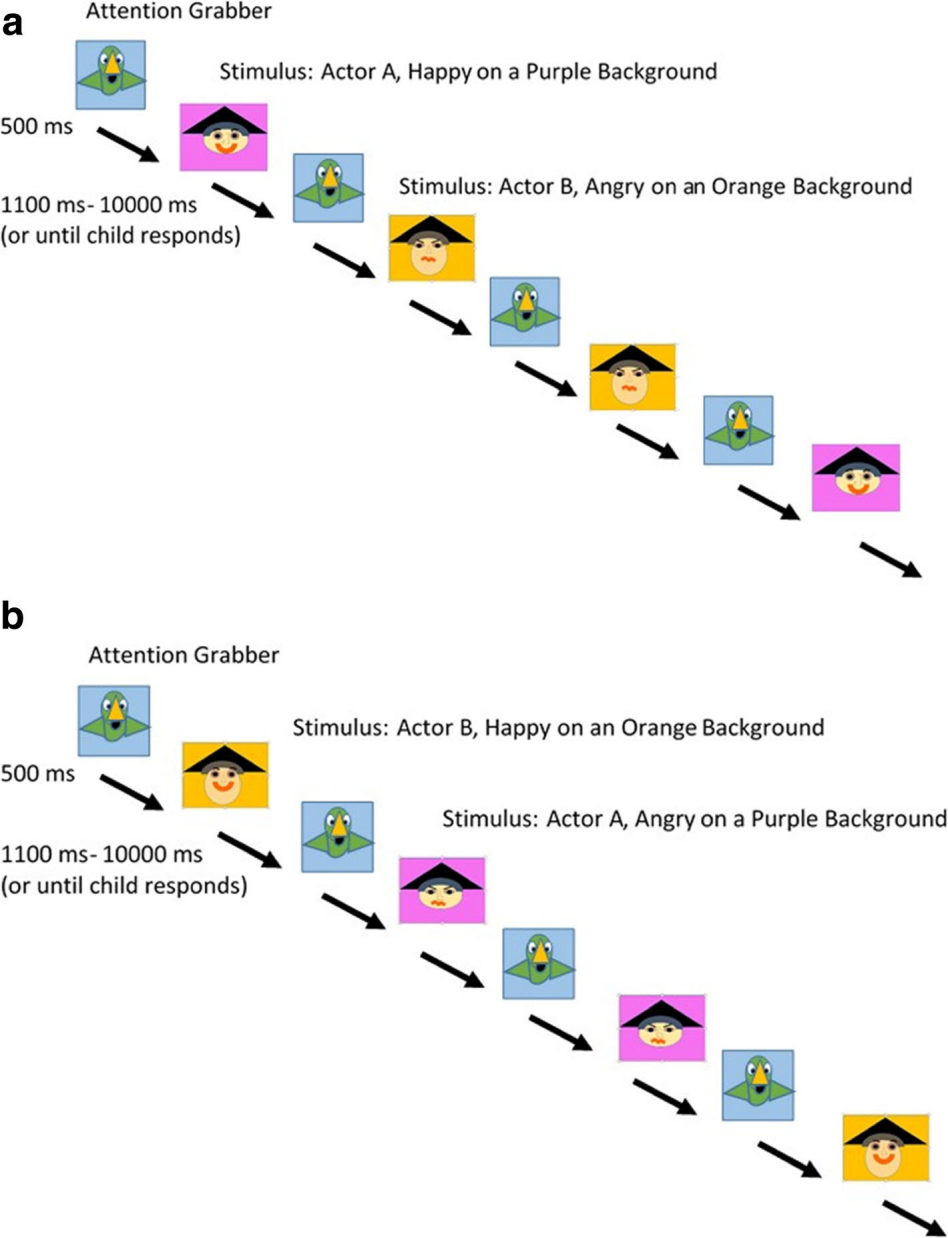
**a** Pre-condition Block. In the Pre-condition, an attention grabber is first presented on the screen. When the experimenter forwards the paradigm, a “squawk” sound is played 500 ms before displaying the happy or angry actor on an orange or purple background. The child was required to push a “happy” or “angry” button on the keyboard depending on the stimulus. Actor A was depicted looking happy and Actor B was depicted looking angry. Depicted images and colors are for presentation purposes only. The attention grabber used was an image from a popular children’s TV; the faces used were from the NimStim, though these stimuli are not presented in this manuscript, in keeping with the NimStim authors’ instructions to limit use for testing purposes. **b** Post-condition Block. The Post-condition is similar to the Pre-condition. However, the actors’ expressions are now swapped for the majority of the 78 post-condition trials. That is, for most trials the actor that previously appeared angry (here, Actor B) was shown looking happy and the actor that previously looked happy (here, Actor A) was shown looking angry. However, to maintain task demands, the pre-condition emotional expressions were displayed 12 times during the post-condition (4 times per block). Depicted images and colors are for presentation purposes only. The attention grabber used was an image from a popular children’s TV; the faces used were from the NimStim, though in keeping with the NimStim authors’ instructions to limit use for testing purposes NimStim images are not presented here

Both the pre- and post- switch portions of the task required attention to, and the perception of, emotional expressions. However, amongst children who formed associations between actors, emotional expressions, and backgrounds during the pre-switch phase, the post-switch phase was expected to require the inhibition of irrelevant exogenous information. Likewise during post-switch, amongst children who formed associations, the task was expected to require the management of conflict resulting from the difference in the current stimuli and the previously learned information (i.e., the expression-actor-color pairing). See Fig. 2a & b.

The paradigm was manually forwarded by an experimenter to ensure that the child was looking at the screen before the stimulus appeared. Each stimulus was presented for a minimum of 1100 milliseconds and up to 10,000 milliseconds or until the child responded. Stimuli were separated by a 500 msec inter-stimulus interval.

The experiment was conducted in a well-lit room and an experimenter was with the child in the room to provide instructions. The experimenter sat behind the child at the child’s 7 o’clock when the test trials began but did not help or guide the child in anyway during the test trials.

### Event related potential data collection and processing

**Fig. 3:**
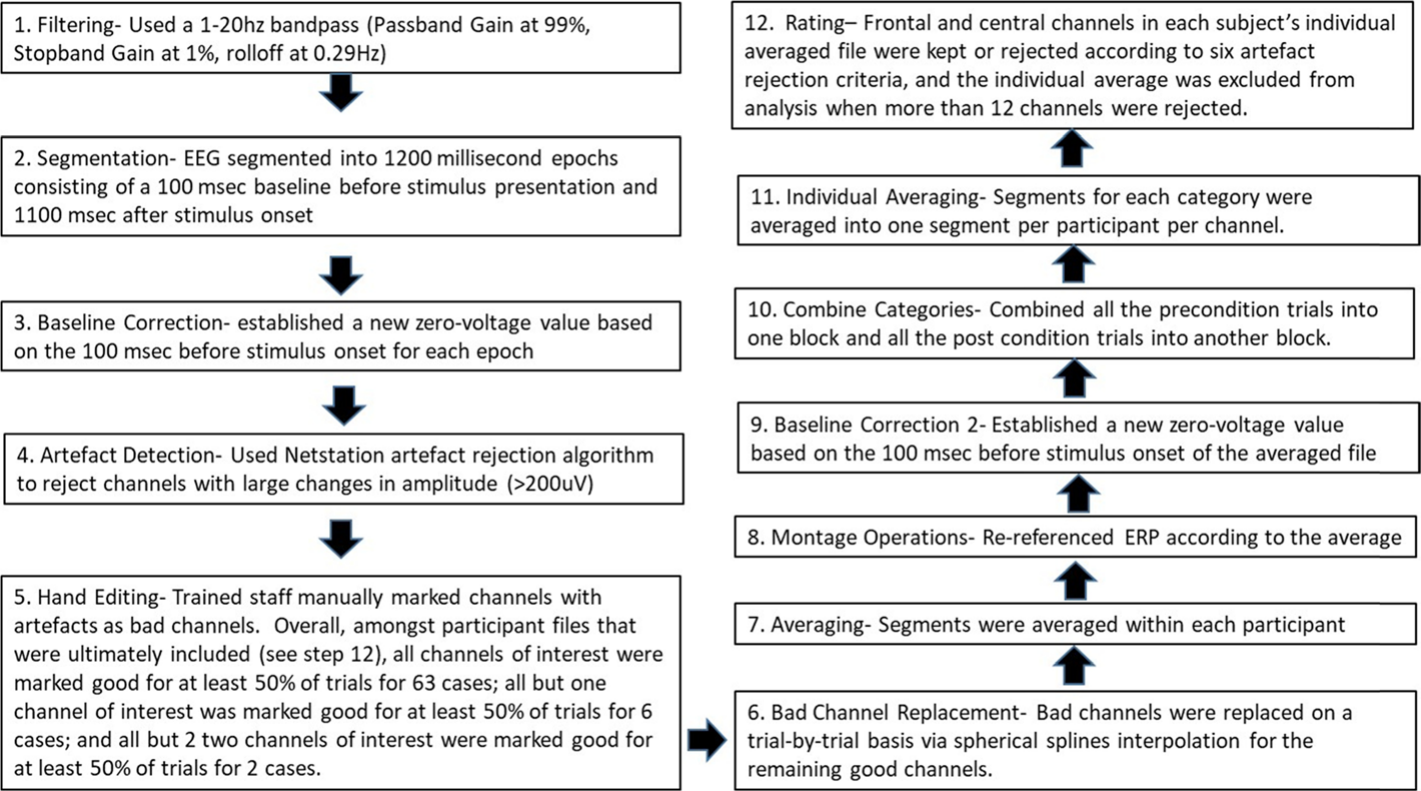
The electrophysiology data processing steps before statistical extraction

ERP was recorded using an EGI Dense Array EEG 300 system with a 0.1–100 Hz filter, initially referenced to the Vertex. A 128-channel geodesic hydrocel routine (sponge) net, without eye electrodes, was used during the experiment. Participants’ electroencephalogram (EEG) recordings were processed using Netstation 4.5.1 software (See Fig. 3). Extraction windows were determined by reviewing both the grand average, as well as individual files so as to capture variation across individuals and across the 44 included frontal and central channels (see Additional file 1: Table S1 for a complete listing as well as Additional file 1: Figure S1). Extraction ranges for the N1, P2, and N2 were, respectively as follows: 62–206 msec, 197–341 msec, and 312–504 msec. These are similar to the timings identified in 4–8 year olds (e.g., initial negative deflection = 92–176; positive deflection = 250; N2 = 350, [20]; N2 = 300–500, [21]). Components were quantified by peak amplitudes. In keeping with studies of young children, we focused upon frontal and central channels (e.g., [20, 21]). Included individual averages were comprised of an average of 37.92 pre-switch (76%; range: 20–49) and 45.23 post-switch (69%; range: 22–26) trials.

### Statistical analyses

#### Task performance

Differences in pre- and post- switch blocks behavioral performance (i.e., accuracy and reaction time) were examined. First, we compared behavioral data in the pre- and post- switch conditions. We expected that children who experienced cognitive conflict during the post-switch phase would exhibit less accuracy and slower reaction times during this latter condition [42]. These analyses were conducted via a Repeated Measures ANCOVA, with gender and antenatal anxiety serving as covariates and pre-versus-post performance serving as the within subjects “Condition” variable. In addition we controlled for whether or not the children passed pre-switch (i.e., 75% or more accuracy). Children who did not pass pre-switch may have had difficulties perceiving emotions, but they may also simply not have understood the task’s instructions. Identical analyses compared the N1, P2_N1 and N2_P2 components during the pre-and post-switch conditions. Neither behavioral nor electrophysiological analyses included data from the 12 trials where pre-switch stimuli were presented in the post-switch blocks.

#### Antenatal anxiety and post-condition ERP’s

In cases where ANCOVA’s revealed significant differences between pre- and post- conditions, relations were further re-explored. In such cases, antenatal maternal anxiety was entered as a predictor variable in a regression where relevant ERP activity served as the outcome variable. These linear regressions adjusted for gender as well as possible confounders of pass-fail status and pre-condition. Given concerns about statistical power, no other potentially relevant covariates were included in our models. Such covariates were screened, and none (i.e., maternal age, household income, maternal education, ethnicity, child age at test) significantly correlated with both maternal mental health and child electrophysiology (see Additional file 1: Table S2).

Next, to examine the potential moderating role of gender, we performed very similar analyses. The difference in this set of analyses was that rather than control for gender, we treated antenatal anxiety, gender, and their interaction as predictors.

Subsequently, to address specificity, identical regressions were repeated examining depression, rather than anxiety, symptomatology. Then, to determine whether any of these observed findings would be better explained via associations with postnatal maternal mental health, we repeated the models containing antenatal maternal mental health symptoms, gender, pre-condition amplitude, and pre-switch passing status, additionally adjusting for postnatal maternal anxiety/depression symptoms. This adjustment was done in a separate step to guard against possible spurious associations arising from adjustment of mediator-colliders [43].

## Results

### Task statistics

#### Descriptive statistics and comparisons of included versus excluded dyads

Amongst the 479 singleton-born preschoolers who attended the laboratory visit, those providing artefact free data (hereafter “ERP+”) were from families with slightly greater household income than were those who did not (hereafter “ERP-,”), t(446) = 2.53, *p* = .012. There were no significant differences between the ERP+ and ERP- groups with regards to maternal education. In addition, the ERP+ children were very slightly but significantly older (i.e., mean difference in age of ~ 8 days), t (477) = 2.24, *p* = 0.025. There were no differences in maternal age, ethnicity, or child gender.

ERP+ preschoolers had mothers who indicated lower levels of antenatal mental health symptoms than those who did not, t (457) _Anxiety_ = − 2.81, *p* = 0.005; t (465) _Depression_ = − 1.98, *p* = 0.048. No significant differences were observed with regards to postnatal maternal mental health.

The ERP+ (*n* = 71) group also did not differ from a subset of the ERP- group (*n* = 33) comprised of children who met task behavioral expectations, but were excluded due to excessive artefacts, with regards to post-switch accuracy, post switch correct reaction time and pre-switch accuracy, though the correct reaction times of the ERP+ group were marginally faster than those of the ERP-group (ERP+: M = 1786.49 msec, SD = 722.35 msec; ERP-: 2109.21 msec, SD = 942.29 msec, t(102) = − 1.92, *p* = 0.06).

Additional statistics, as well as means/frequencies for the ERP+ and ERP- groups can be found in Table 1.

**Table 1:** Characteristics of the ERP+ and ERP- Groups

Continuous Variables								
	ERP+ Group		ERP- Group					
	Mean	SD	Mean	SD		DF	T or F Statistic	
Maternal Age	31.48	5.23	30.82	5.13		477	0.10	
Child Age in Days	1264.85	30.01	1256.70	27.93		477	2.24^*^	
Household Income Category	4.0	1.07	3.63	1.10		446	2.53^*^	
Maternal Education Category	3.19	0.89	3.01	0.92		470	1.48	
Maternal Antenatal Anxiety	31.79	9.13	35.47	10.01		457	2.81^**^	
Maternal Antenatal Depression	6.80	3.74	7.91	4.39		465	1.98^*^	
Maternal Postnatal Anxiety	6.30	5.39	6.58	4.68		348	−0.77	
Maternal Postnatal Depression	33.34	9.89	34.41	9.81		344	−0.42	
% Correct Pre-Switch	79.31	27.81	76.30	20.52		102	0.56	
% Correct Post-Switch	79.25	28.69	73.51	18.34		102	1.05	
Correct Reaction Time Pre Switch	1786.49	722.35	2109.21	942.29		102	1.92^†^	
Correct Reaction Time Post Switch	1957.15	510.15	2068.13	628.06		102	0.96,	
Categorical Variables								
	ERP+ Group			ERP-Group				
	Females (%)	Males (%)		Females (%)	Males (%)		DF	*χ*^2^
Gender	29(41%)	42(59%)		191 (47%)	217(53%)		1	.87
	ERP+ Group			ERP-Group			DF	*χ*^2^
Ethnicity	Chinese (%)	Malay (%)	Indian (%)	Chinese (%)	Malay (%)	Indian (%)		
	40(56%)	19(29%)	11(15%)	226(57%)	115(27%)	60(16%)	2	.08

Ethnicity data was not available for 1 ERP+ and 7 ERP- dyads. Ethnicity descriptives and statistics do not include these missing cases

^**^p < .01, ^*^p < .05, ^†^*p* < .10

#### Pre- vs post- condition behavioral data in the included sample

As reported in Additional file 1: Table S3, accounting for antenatal anxiety, gender, and whether or not the child passed pre-switch, no significant differences were observed with either accuracy or correct reaction times across pre and post conditions, nor were any interactions between gender/antenatal anxiety/failure of pre-switch and pre-post performance observed.

#### Pre- vs post- condition electrophysiological data (see Table 2)

When considering whether or not children passed pre-switch, as well as anxiety and gender, significant differences were observed between the N1 amplitude in the pre and post condition (F (1, 64) = 7.74, *p* < 0.01), as well as for the P2_N1 amplitude in the pre and post condition (F (1, 64) = 6.27, *p* < 0.05), but no significant differences were observed for N2_P2 amplitude. In addition, both the N1 amplitude pre-post difference (F (1, 64) = 5.62, *p* < .05) and the P2_N1 amplitude pre-post difference (F (1, 64) = 6.24, *p* < .05)) were significantly moderated by anxiety. The P2_N1 amplitude pre-post difference was also significantly moderated by gender, F (1, 64) = 5.58, *p* < 0.05).

**Table 2:** Comparison of Pre and Post ERP components accounting for antenatal maternal anxiety and gender

	Pre-switch condition (*n* = 68; 41 male & 27 female)				Post-switch condition (*n* = 68; 41 male & 27 female)					
	*M*_*female*_	*SD*_*female*_	*M*_*male*_	*SD*_*male*_	*M*_*female*_	*SD*_*female*_	*M*_*male*_	*SD*_*male*_	F	df
N1 Amplitude	−7.72	2.75	−7.30	2.69	−8.58	3.09	−7.11	2.25		
N1 Amplitude Pre-Post									7.74**	1,64
Antenatal Anxiety X N1 Amplitude Pre-Post									5.62*	1,64
Gender X N1 Amplitude Pre-Post									3.72*	1,64
Failed Pre-switch X N1 Amplitude Pre-Post									1.32	1,64
P2_N1 Amplitude	8.700	3.44	8.49	4.34	10.18	4.62	8.05	4.00		
P2_N1 Amplitude Pre-Post									6.27*	1,64
Antenatal Anxiety X P2_N1 Amplitude Pre-Post									6.24*	1,64
Gender X P2_N1 Amplitude Pre-Post									5.58*	1,64
Failed Pre-switch X N1 Amplitude Pre-Post									1.16	1,64
N2_P2 Amplitude	−9.18	4.14	−9.01	4.59	−10.20	4.45	−8.66	4.17		
N2_P2 Amplitude Pre-Post									1.49	1,64
Antenatal Anxiety X N2_P2 Amplitude Pre-Post									1.35	1,64
Gender X N2_P2 Amplitude Pre-Post									2.37	1,64
Failed Pre-switch X N1 Amplitude Pre-Post									.22	1,64

Means and Standard Deviations are not adjusted for maternal anxiety or pre-switch pass status

*p < 0.05, **p < 0.01, ***p < 0.001

### Antenatal anxiety and post condition ERP’s (see Table 3 and Figs. 4, 5, 6)

As noted, the N1 and P2_N1 amplitudes significantly differed between pre-and post- conditions. Therefore, we further explored anxiety’s relation to the N1 post condition and the P2_N1 post condition in a series of regression analyses, reported in Table 3 (See Additional file 1: Table S4, for correlations between mental health and the N1-PRE, P2_N1-PRE, N1-POST, and separately P2_N1-POST).

**Table 3:** Regression results for N1, and P2_N1 amplitudes: unstandardized beta (B) and standardized beta (Std. Beta)

Antenatal maternal mental health: N1 POST Amplitude							
	B (Std Error)		Std Beta		B (Std Error)		Std Beta
*Anxiety (n = 68)*				*Depression (n = 69)*			
Antenatal anxiety	.11***	(.03)	.38***	Antenatal depression	.25**	(.08)	.35**
Gender	−1.84**	(.60)	−.34**	Gender	−1.54*	(.60)	−.28*
Pre-condition	.25*	(.11)	.25*	Pre-condition	.29*	(.11)	.29*
Pass Pre-Switch	−.08	(.73)	−.01	Pass Pre-Switch	.05	(.75)	.01
Antenatal maternal mental health: N1 POST Amplitude accounting for postnatal maternal mental health (Final Block)							
	B (Std Error)		Std Beta		B (Std Error)		Std Beta
*Anxiety (n = 58)*				*Depression (n = 59)*			
Antenatal anxiety	.15**	(.04)	.48***	Antenatal depression	.35**	(.11)	.43**
Gender	−1.82**	(.65)	−.33**	Gender	−1.56*	(.65)	−.28*
Pre-condition	.14	(.13)	.14	Pre-condition	.22†	(.12)	.22†
Pass Pre Switch	.16	(.86)	.02	Pass Pre Switch	.15	(.88)	.02
Postnatal Anxiety	−.05	(.04)	−.18	Postnatal Depression	−.03	(.07)	−.06
Antenatal maternal mental health: NI POST Amplitude gender moderation (Final Block)							
	B (Std Error)		Std Beta		B (Std Error)		Std Beta
*Anxiety (n = 68)*				*Depression (n = 69)*			
Antenatal anxiety	.12***	.03	.40***	Antenatal depression	.23**	.08	.32**
Gender	−1.97**	.59	−.36**	Gender	−1.58**	.56	−.29**
Pre-condition	.24*	.11	.24*	Pre-condition	.30**	.11	.30**
Pass Pre Switch	.02	.72	.00	Pass Pre Switch	.02	.74	.00
Antenatal anxiety X Gender	.13†	.07	.20†	Antenatal depression X Gender	.31*	.15	.21*
Antenatal maternal mental health: P2_N1 POST Amplitude							
	B (Std Error)		Std Beta		B (Std Error)		Std Beta
*Anxiety (n = 68)*				*Depression (n = 69)*			
Antenatal anxiety	−.16***	(.05)	−.34***	Antenatal depression	−.31	(.12)	−.27*
Gender	2.65**	(.89)	.30**	Gender	2.19	(.90)	.25*
Pre-condition	.46***	(.11)	.42***	Pre-condition	.47	(.11)	.42***
Pass Pre-Switch	−1.79†	(1.08)	−.17	Pass Pre-Switch	−1.93	(1.13)	−.18†
Antenatal maternal mental health: P2_N1 POST Amplitude accounting for postnatal maternal mental health (Final Block)							
	B (Std Error)		Std Beta		B (Std Error)		Std Beta
*Anxiety (n = 58)*				*Depression (n = 59)*			
Antenatal anxiety	−.22***	.06	−.45***	Antenatal depression	−.52**	(.16)	−.40**
Gender	2.44*	.97	.28*	Gender	2.05*	(.98)	.23*
Pre-condition	.39**	.13	.34**	Pre-condition	.42**	(.13)	.37**
Pass Pre Switch	−2.02	1.25	−.18	Pass Pre Switch	− 2.22†	(1.28)	−.20†
Postnatal anxiety	.07	.06	.16	Postnatal depression	.13	(.10)	.15
Antenatal maternal mental health: P2_N1 POST Amplitude gender moderation (Final Block)							
	B (Std Error)		Std Beta		B (Std Error)		Std Beta
*Anxiety (n = 68)*				*Depression (n = 69)*			
Antenatal anxiety	−.17	(.05)	−.36***	Antenatal depression	−.29	(.12)	−.24*
Gender	2.81	(.89)	.32**	Gender	2.24	(.90)	.25*
Pre-condition	.45	(.11)	.42***	Pre-condition	.47	(.11)	.42***
Pass Pre Switch	−1.91	(1.07)	−.18†	Pass Pre Switch	−1.91	(1.12)	−.18†
Antenatal anxiety X Gender	−.16	.10	−.16	Antenatal depression X Gender	−.37	.24	−.16

^†^*p* < .10 *p < 0.05, **p < 0.01, ***p < 0.001

**Fig. 4:**
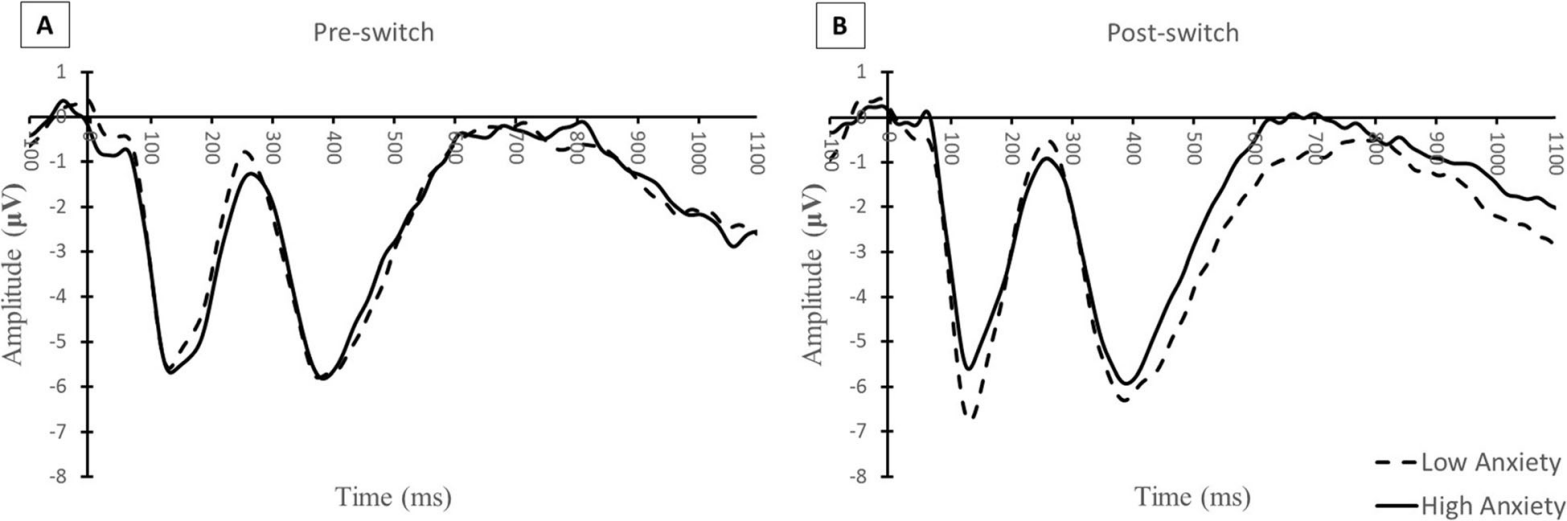
Comparison of Anxiety Grand Averages. A side by side comparison of the grand averages (average of frontal and central electrodes) in the pre-condition (**a**) and post-condition (**b**) sorted by two groups of anxiety scores – 20 to 30 (*n* = 34) and 30 to 60 (*n* = 34)

**Fig. 5:**
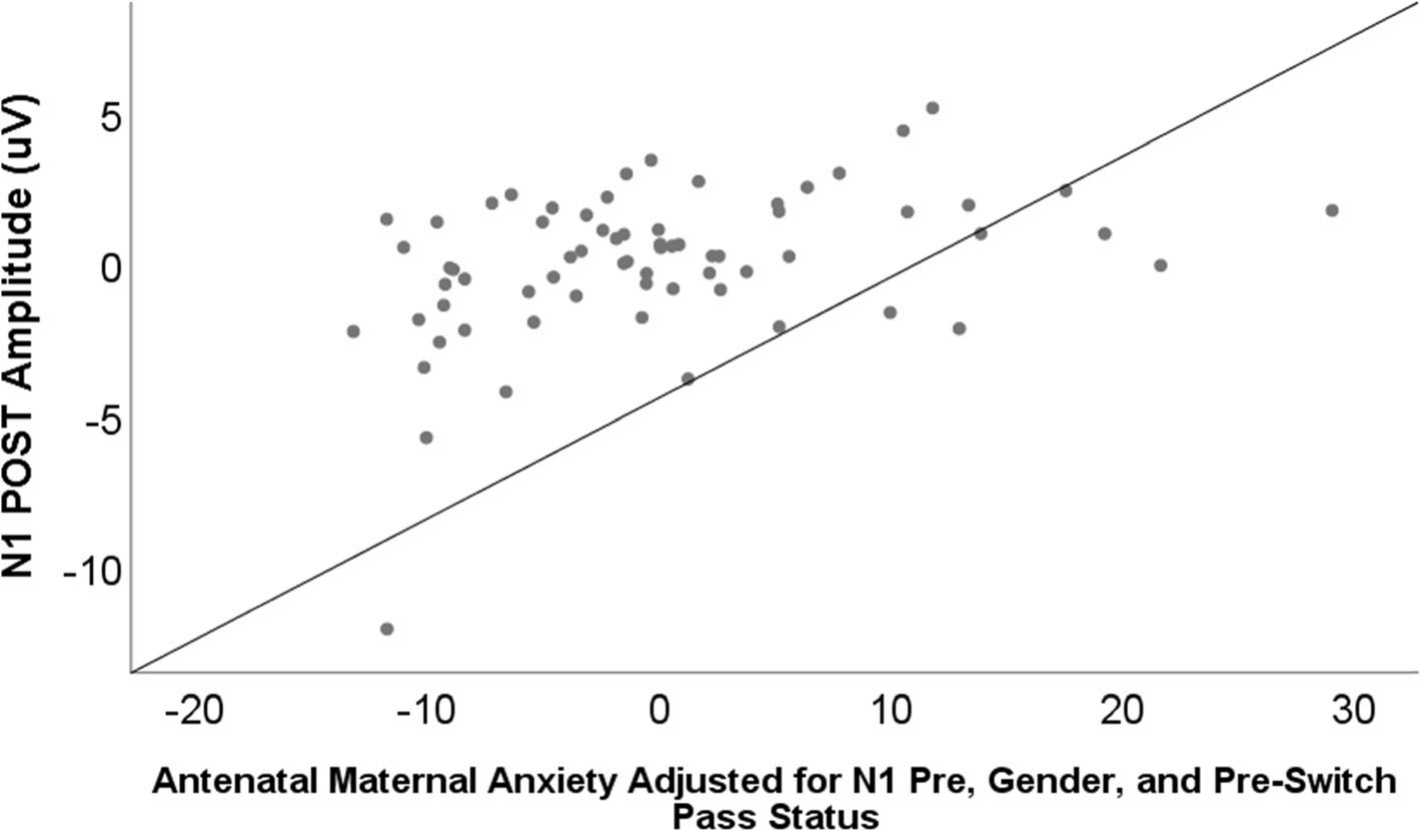
Scatterplot of the relation between antenatal maternal anxiety and N1 amplitude at Post-Switch, B = 11, SE = 0.03, *B =* 0.38, *p* = 0.001. When the one outlying value (included in the current graph) was removed from the sample, results remained significant, B = .08, SE = 0.03, *p* = .002

**Fig. 6:**
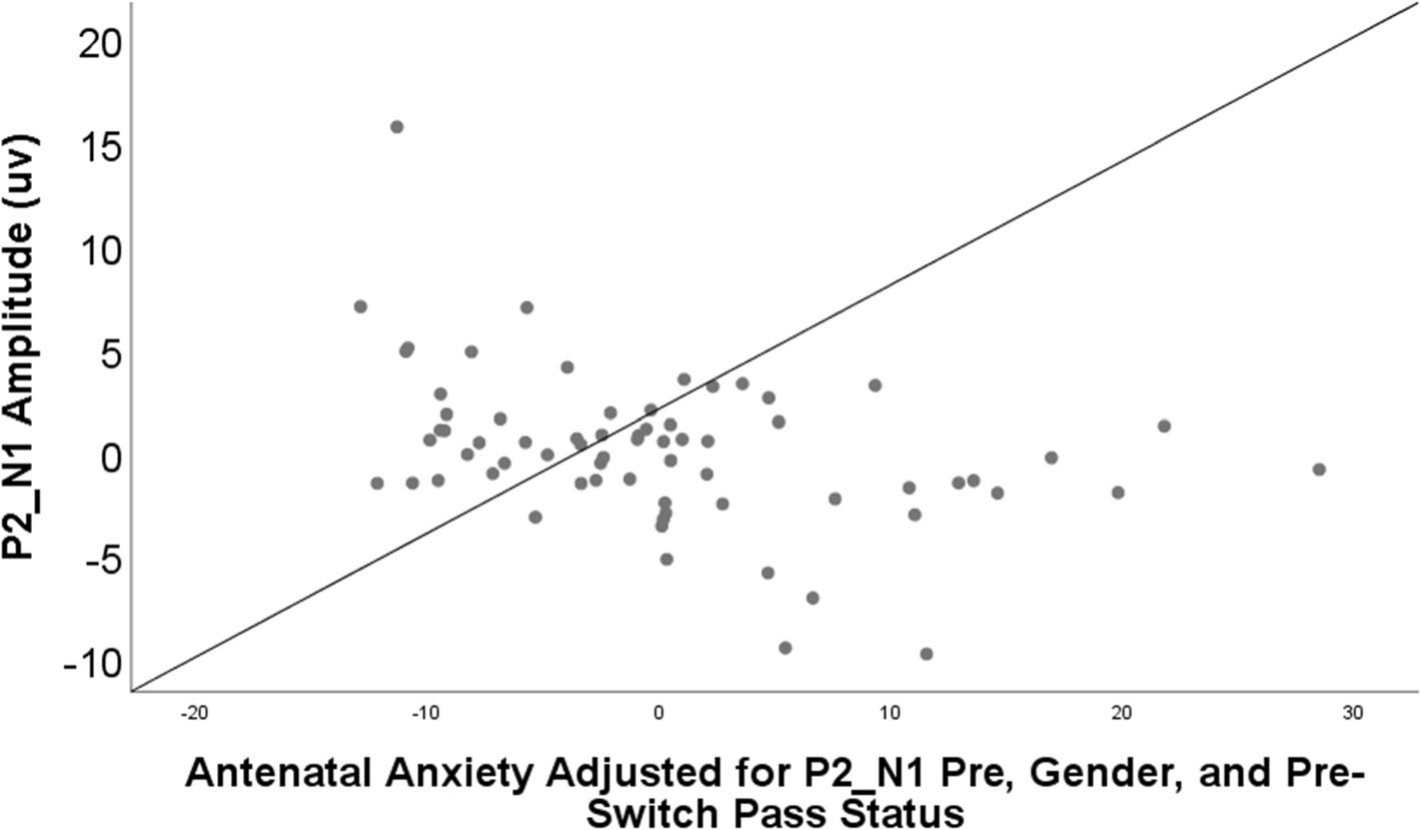
Scatterplot of the relation between antenatal maternal anxiety and the P2_N1 amplitude at Post-Switch, B = −0.16, SE = 0.05, *B = −*0.34, *p* = 0.001. When the one outlying value (included in the current graph) was removed from the sample, results remained significant, B = −.13, SE = 0.04, *B* = −.30, *p* = .004

Both maternal anxiety and maternal depression significantly associated with “smaller” (less negative) N1-POST amplitudes, (Anxiety: *B = 0.38, p* < .001; Depression: *B* = 0.35, *p* < .001). These relations were marginally (in the case of anxiety) and significantly (in the case of depression) moderated by gender (Anxiety*Gender: *B = 0.20, p*<. 10; Depression*Gender: *B* = 0.21, *p* < .05). Both antenatal anxiety and depressive symptoms remained significant predictors of N1-POST when postnatal maternal mental health was considered (Anxiety: *B = 0.48, p* < .001; Depression: *B* = 0.42, *p* < .001).

In addition, both maternal anxiety and maternal depression significantly associated with smaller (less positive) P2_N1-POST amplitudes (Anxiety: *B = − 0.34, p* < .001; Depression: *B* = − 0.27, *p* < .05. Though gender was also significantly associated it did not moderate relations with P2_N1-POST amplitudes. Both antenatal anxiety and depressive symptoms remained significant predictors of P2_N1-POST when postnatal maternal mental health was considered (Anxiety: *B = 0.45, p* < .001; Depression: *B* = 0.40, *p* < .01).

Because one case was an outlier from the regression mean in all our analyses, we repeated the regressions after removing that case. Regardless of whether we included maternal postnatal anxiety into the models, or concurrently examined maternal anxiety x gender, the main effect of maternal antenatal anxiety remained significantly predictive of the N1 and P2_N1, with *p* values ranging from *p* < 0.001 to *p* = 0.004. Likewise, regardless of whether we included maternal postnatal depression in our models, or included the interactive effect of depression x gender, the main effect of maternal antenatal depression remained a significant (i.e., in four models p’s ranged from 0.007 to 0.016) or marginal (i.e., in two models p was <.10) predictor of the N1 and P2_N1. In contrast, after removing the one outlying case, the interactive effects of maternal mental health and gender upon the N1 became non-significant (antenatal anxiety x gender: *p* = 0.53; antenatal depression x gender: *p* = 0.44), and remained non-significant with regards to the P2_N1.

## Discussion

Despite maternal anxiety’s prevalence and its import to child outcomes, to our knowledge, this is the first investigation of maternal antenatal anxiety symptomatology and performance during a preschool electrophysiology task designed to tap executive functioning, as well as attention, emotion perception, and memory formation. We observed significant associations between antenatal maternal anxiety symptoms and preschoolers’ neurophysiology, which remained after adjusting for postnatal influences. This echoes findings from a large-scale examination of maternal antenatal and postnatal anxiety and other aspects of cognitive functioning [5]. Likewise, we observed similar associations between antenatal maternal depression symptoms and preschool functioning, despite relatively low average scores for maternal antenatal depression in the current sample.

Notable associations occurred between maternal mental health and both the N1-POST and N1_P2-POST amplitudes. Higher levels of antenatal anxiety, and depression, predicted less N1-POST activity, or less downward fluctuation in this negative-going early component, often associated with sensory discrimination and exogenous inhibition. Past research suggests exogenous inhibition may not be affected in older offspring of mothers with antenatal anxiety [7], though work with infants finds antenatal anxiety predictive of alterations in relatively early stages of information processing including those indicative of attention [23, 24]. In accordance with these findings from infants, here in our sample of preschoolers, we additional observed higher antenatal maternal anxiety and depression associated with smaller P2 amplitudes, perhaps indicating differential attention to the emotional faces. For example, social anxiety in children has previously been found associated with lower P2 amplitudes from frontal channels in response to pictures of angry and neutral NimStim faces [44]. Still, and of note, in the current research, significant correlations were only observed between the maternal mental health variable and post, but not pre, switch components. Were observed differences entirely due to variation in emotional salience and/or perception, relations should have been observed with both pre- and post-switch blocks. Alternatively, then, our findings might also suggest that those exposed to higher levels of antenatal maternal mental health symptoms found the task to be *easier*, requiring less attentional capacity. Why might this be the case?

One post-hoc explanation is that preschoolers born to mothers who experienced higher levels of maternal mental health symptomatology may have approached the task differently, and thus had less need for attentional processing as well as exogenous inhibition during the post-switch phase. Although work in other fields suggests that elements of anticipatory control may be observed beginning in infancy [45], ERP research suggests that young children may not exhibit attentional anticipatory control unless required to do so, instead relying on reactive control. The P2 may index attention to salient information as well as proactive interference, arising from a prior stimulus-associated response—with its magnitude influenced by the extent of the prior association. If children formed a strong association between the context (i.e., actor identity and background color), expression, and left-versus-right button press response, then following the switch, the prior context-response association may have been considered more salient and/or created proactive interference for children as they assessed the stimuli. If, children born to mothers higher on anxiety did not (or did not fully) form such associations, then the significance of the stimuli as well as any proactive interference would be expected to decline, and their P2s might have been smaller in magnitude than those born to mothers lower on anxiety. Likewise, more negative N1’s are typically associated with greater difficulty [46]. If preschoolers (born to mothers low on antenatal anxiety) were better at forming associations between the colors-faces-and-actors during the pre-switch condition, they would have, de facto, have had more need for exogenous inhibition during the post-switch, and so more pronounced N1-POSTs. Indeed, within GUSTO, antenatal anxiety associates with infant hippocampal development [47] and worsened memory for associations between pairs [48].

Alternatively, it is also possible that the children who were high on antenatal anxiety were simply less distracted by extraneous details, focusing more on the emotions, and so showing less conflict or attention. However, this does not seem likely as significant simple correlations between maternal mental health and pre-switch N1 and P2_N1 components were not observed. As such it also seems unlikely that the current findings simply reflect a lack of overall task engagement.

Despite its novelty, the current work had a number of limitations. Given the paucity of similar preschool research, and because we expected our final sample would be limited in size, we created a task that we hoped would tap cognitive flexibility/inhibition but that also was achievable by children of this age and incorporated multiple processes likely impacted by anxiety. This approach increased feasibility and ecological validity and allowed us the greatest chance of avoiding Type II errors. However, it limits our understanding of the exact cognitive mechanism behind the observed association. In addition, it also limits the extent to which we can compare our findings with those observed in older offspring of mothers high on antenatal anxiety. Work with older offspring suggests relations with endogenous forms of cognitive inhibition (i.e., the N2). Here we did not observe such effects. This may not be surprising as work examining the N2 in 4–8 year olds suggests that the N2 effect may only be observed in children older than 6 years of age [20]. Furthermore, our pattern of findings suggests that for some children, this task may not even have measured executive control. Likewise, though we suspect that our between group N1 and P2 differences may have been influenced by differences in the formation of associations between expressions, actors, colors, and responses, we do not have enough post-switch trials to further test this explanation. To better assess this idea, future work should determine whether, as would be expected [49], N1 and P2 amplitudes decrease across repeat post-switch trials as children update associative memory with the post-switch color-actor-expression associations. Likewise it should examine whether N1 amplitudes become less pronounced as extraneous aspects of the stimuli become less salient.

Second, the large amount of data loss cannot be ignored, and may have been further minimized by the use of more technologically advanced ERP processing pipelines including newly freely available platforms such as HAPPE [50]. Yet, our sample of 71 three-and-a-half year olds is as large or larger than other similar preschool ERP investigations of executive functioning (e.g., *n* = 50 [51]; *n* = 27 [52]). Moreover, the most similar research, which has been conducted with not only 3 year olds but also older preschoolers who may be more compliant, also exhibits somewhat high levels of data loss (e.g. 30% lost in three-to-four-and-a-half year olds, [21]; 45% lost in 3-to-5 year old control subjects, [51]; 25% lost in four-to-six-year-olds, [52]). In the current research, amongst children who took part in the task and fulfilled behavioral criteria, about 25% were excluded due to excessive artefacts. The majority of data were “lost” due to the parent’s or preschooler’s refusal to take part in the task (*n* = 174) or because the preschooler did not meet behavioral expectations during the task (*n* = 182). It is difficult to know whether similar refusal rates occur in other developmental ERP research: unlike what occurs in many projects, here, participants did not come to the laboratory specifically to take part in an ERP experiment, but rather to participate in a general cognitive-emotional follow up session for 3 year olds participating in a cohort study. That is, the sample was not pre-selected for ERP interest, nor was performing ERP the only goal of the visit. Indeed, this may have resulted in a more representative sample than what is typically observed- with demographics indicating that the ERP+ and ERP- groups were similar with regards to maternal education. Furthermore, although the groups did show differences with regards to household income, even the “wealthier” ERP+ group had mean incomes and educational levels that were in keeping with those observed in Singapore as a whole. This is in contrast to other ERP samples with levels of maternal education that exceed the population median. Nevertheless, although mean antenatal mental health scores amongst both ERP+ and ERP- mothers were below STAI and EPDS clinical screening “cutoffs” [53], ERP- mothers reported significantly higher scores. Future research may wish to specifically target children whose mothers screened high for antenatal maternal mental health problems to determine whether the current results differ in populations at greater risk. The inclusion of a broader participant pool may also allow for a better understanding of the moderating role of gender at this developmental stage. Work with older children and adolescents [9, 28] reports larger effects in male than female offspring. Whilst our initial findings suggested that maternal mental health differentially impacted female and male preschool offspring electrophysiology, after removing an outlier, the maternal mental health by gender interactions did not remain significant. Larger more inclusive studies will be able to better determine whether our initial findings concerning an interactive role of gender were spurious.

## Conclusions

We observed antenatal maternal anxiety and depression symptoms related to neurophysiology. These effects were not attenuated by postnatal maternal mental health. This electrophysiological study, then, echoes research examining antenatal mental health and neonatal brain development [27, 54, 55], and underscores the importance of interventions to improve maternal psychological well-being prior to or during the antenatal period. Moreover, our findings suggest the potential need for early life cognitive-emotional intervention-prevention programs targeted to offspring of mothers who experienced depression and anxiety symptoms during pregnancy. Such programs might focus on associative memory, exogenous sensory inhibition, attention, and/or emotional processing difficulties.

## Supplementary information

**Additional file 1: Table S1.** List of Included Electrodes by Hemisphere and Region. **Table S2.** Relations between Covariates, Antenatal Maternal Mental Health, and Preschool Electrophysiology. **Table S3.** Task Behavioral Performance. **Table S4.** Correlations between Maternal Mental Health and ERP Variables. **Figure S1.** Topography in the 71 ERP+ sample.

## Data Availability

De-identified datasets analyzed during the current study are available from the corresponding author on reasonable request.

## Abbreviations

ERP: Event related potential
ERP-: GUSTO children who attended the preschool visit and did not provide artefact free ERP data
ERP+: GUSTO children attended the preschool visit and provided artefact free ERP data
GUSTO: Growing Up in Singapore towards Healthy Outcomes cohort study
N1: Negative 1 Component
N2: Negative 2 Component
P2: Positive 2 Component
POST: Post-switch condition of the experimental task
PRE: Pre-switch condition of the experimental task
