# Supplementary material for: Maternal antenatal anxiety and electrophysiological functioning amongst a sub-set of preschoolers participating in the GUSTO cohort
Source: BMC Psychiatry. 2020 Feb 12;20:62. doi: 10.1186/s12888-020-2454-3 (PMC7017524; doi:10.1186/s12888-020-2454-3)
Supplement: Supplementary file 1 — Additional file 1: Table S1. List of Included Electrodes by Hemisphere and Region. Table S2. Relations between Covariates, Antenatal Maternal Mental Health, and Preschool Electrophysiology. Table S3. Task Behavioral Performance. Table S4. Correlations between Maternal Mental Health and ERP Variables. Figure S1. Topography in the 71 ERP+ sample. [file 12888_2020_2454_MOESM1_ESM.pdf]

Supplementary Table 1. List of Included Electrodes by Hemisphere and Region

---

Frontal Midline Channels (n = 3)

Frontal Midline Channel 15

Frontal Midline Channel 16

Frontal Midline Channel 11

Central Midline Channels (n = 1)

Central Midline Channel 6

Frontal Left Channels (n = 10)

Frontal Left Channel 26

Frontal Left Channel 23

Frontal Left Channel 19

Frontal Left Channel 12

Frontal Left Channel 33

Frontal Left Channel 27

Frontal Left Channel 24

Frontal Left Channel 20

Frontal Left Channel 34

Frontal Left Channel 28

Frontal Right Channels (n = 10)

Frontal Right Channel 2

Frontal Right Channel 3

Frontal Right Channel 4

Frontal Right Channel 5

Frontal Right Channel 122

Frontal Right Channel 123

Frontal Right Channel 124

Frontal Right Channel 118

Frontal Right Channel 116

Frontal Right Channel 117

Central Left Channels (n = 10)

Central Left Channel 7

Central Left Channel 13

Central Left Channel 29

Central Left Channel 35

Central Left Channel 41

Central Left Channel 36

Central Left Channel 30

Central Left Channel 42

Central Left Channel 37

Central Left Channel 31

Central Right Channels (n = 10)

Central Right Channel 112

Central Right Channel 106

Central Right Channel 80

Central Right Channel 105

Central Right Channel 111

Central Right Channel 110

Central Right Channel 104

Central Right Channel 87

Central Right Channel 103

Central Right Channel 93

Supplementary Table 2. Relations between Covariates, Antenatal Maternal Mental Health, and Preschool Electrophysiology

	Maternal Age	Child Age in Days	Household Income	Maternal Highest Education	Ethnicity
N1 Pre	0.11	0.05	-0.07	-0.01	1.55
N1 Post	-0.05	-0.06	-0.19	-0.16	3.86*
P2_N1 Pre	-0.03	0.03	-0.01	-0.02	1.77
P2_N1 Post	0.03	0.02	0.16	0.13	2.384†
N2_P2 Pre	-0.02	0.12	-0.10	-0.03	1.12
N2_P2 Post	-0.06	0.17	-0.15	-0.09	0.970
Antenatal Anxiety	-0.05	-0.03	-0.08	-0.21†	2.73†
Antenatal depression	-0.20†	0.06	-0.01	-0.18	0.08

***p≤ .001, **p≤ .01, *p≤ .05, p≤ .10

Relations examining continuous screened covariates (i.e., maternal age, child age, household income, and maternal education) were performed with Pearson correlations, and the above values reflect Pearson R's. Associations between ethnicity and the maternal mental health/electrophysiological components were assessed via ANOVA, with the above values reflecting the F statistic. N's are as follows: ERP components and maternal age/child age/maternal education, N = 71; ERP components and ethnicity, N = 70; ERP components and household income, N = 66.; antenatal anxiety and maternal age/child age, N = 68; antenatal anxiety and household income, N = 64; antenatal anxiety and maternal education/ethnicity, N = 67; antenatal depression and maternal age/child age, N = 69; antenatal depression and child age, N = 69; antenatal depression and maternal education/ethnicity, N = 68; antenatal depression and household income, n = 66.

Supplementary Table 3. Task Behavioral Performance

Descriptives of Pre and Post Block Behavioral Performance

	n	Preswitch Block		Postswitch Block	
		Mean	SD	Mean	SD
Accuracy (%) All ERP Cases	71	0.87	0.11	0.87	0.13
Correct Reaction Time All ERP Cases	71	1786.49	722.35	1957.15	510.15
Accuracy (%) Antenatal Anxiety Sample	68	0.87	.11	0.86	0.13
Correct Reaction Time Antenatal Anxiety Sample	68	1810.74	728.17	1969.39	517.23

Comparison of Pre and Post Accounting for Anxiety, Gender, & Pres-switch pass versus fail status

**Accuracy**	F	Df	*p*
Pre versus Post Accuracy	.976	1,64	.327
Antenatal Anxiety X Pre versus Post Accuracy	.573	1,64	.452
Gender X Pre versus Post Accuracy	.284	1,64	.596

Pre-switch pass/fail status X Pre versus Post Accuracy	2.322	1,64	.132
**Reaction Time**			
Pre versus Post Correct Reaction Time	.205	1,64	.653
Antenatal Anxiety X Pre versus Post Correct Reaction Time	.022	1,64	.882
Gender X Pre versus Post Correct Reaction Time	.563	1,64	.456
Pre-switch pass/fail status X versus Post Correct Reaction Time	1.463	1,64	.231

Supplementary Table 4. Correlations between Maternal Mental Health and ERP Variables

	N1 PRE	P2_N1 PRE	N2_P2 PRE	N1 POST	P2_N1 POST	N2_P2 POST
Antenatal Anxiety (n = 68)	-0.01	-0.06	0.16	.309**	-.271*	.257*
Antenatal Depression (n = 69)	-0.07	-0.11	0.21†	.290*	-.238*	.259*
Postnatal Anxiety (n = 61)	-0.24†	0.12	-0.02	0.02	-0.01	0.05
Postnatal Depression (n = 61)	-0.18	0.01	0.06	0.07	-0.01	0.02

*$p \leq 0.05$, **$p \leq 0.01$, ***$p \leq 0.00$

Supplementary Figure 1. Topography in the 71 ERP+ sample

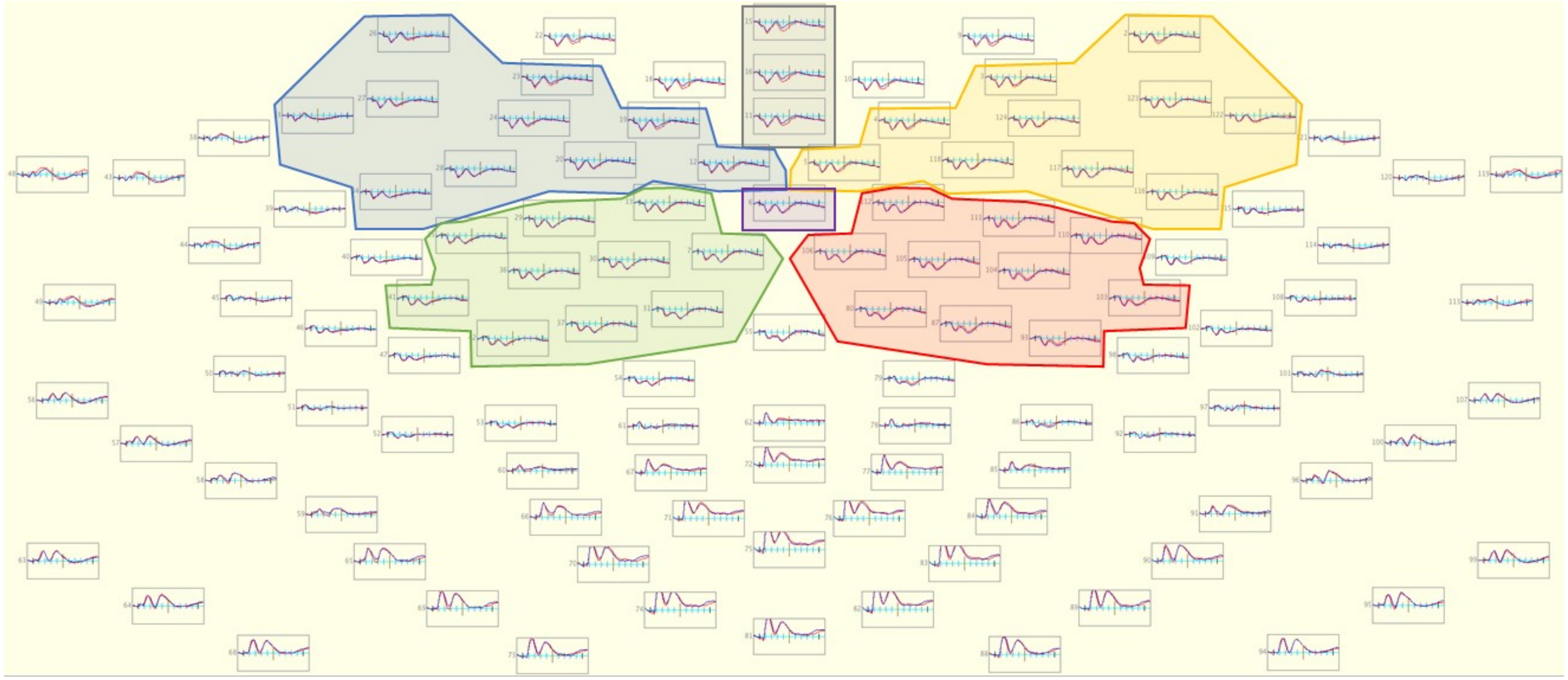

Included channels are outlined in blue (Frontal Left), yellow (Frontal Right), green (Central Left), red (Central Right), and by the boxed regions (Midline Frontal and Midline Central).
